# Supplementary material for: Extracellular Forces Cause the Nucleus to Deform in a Highly Controlled Anisotropic Manner
Source: Sci Rep. 2016 Feb 19;6:21300. doi: 10.1038/srep21300 (PMC4759536; doi:10.1038/srep21300)
Supplement: Supplementary Information [file srep21300-s2.pdf]

Supplementary Information

Extracellular Forces Cause the Nucleus to Deform in a Highly Controlled Anisotropic Manner

Kristina Haase[a], Joan K. L. Macadangdang[a], Claire H. Edrington[a], Charles M. Cuerrier[a], Sebastian Hadjiantoniou[a,b], James L. Harden[a,c], Ilona S. Skerjanc[d], and *Andrew E. Pelling[a,b,e]

[a]*Centre for Interdisciplinary NanoPhysics, Department of Physics, MacDonald Hall, 150 Louis Pasteur*
[b]*Department of Biology, Gendron Hall, 30 Marie Curie*
[c]*Ottawa Institute of Systems Biology, Roger Guindon Hall, 451 Smyth Road*
[d]*Department of Biochemistry, Microbiology & Immunology, Roger Guindon Hall, 451 Smyth Road*
[e]*Institute for Science Society and Policy, Simard Hall, 60 University*
University of Ottawa, Ottawa, ON, K1N 6N5, Canada.

*Correspondence To:
Andrew E. Pelling
150 Louis Pasteur
MacDonald Hall
University of Ottawa
Ottawa, ON K1N 6N5
Canada
Tel. +1 613 562 5800 Ext 6965
Fax. +1 613 562 5190
Email: a@pellinglab.net
Web: http://www.pellinglab.net

Materials and methods

Cell culture.

Mouse (NIH3T3) and human (HFF) fibroblasts, canine (MDCK), and human (HeLa) epithelial cells, as well as mouse muscle (C2C12) cells, were cultured in Dulbecco's Modified Eagle's Medium (DMEM, Hyclone, Logan, Utah, USA) supplemented with 10% fetal bovine serum (FBS) and 1% penicillin/streptomycin (P/S) at 37°C and 5% CO_2. Hampster (CHO) cells were cultured in DME/F-12 with 10% FBS and 1% P/S (Hyclone, Logan, Utah, USA). Cells used in experiments were grown on 35 mm glass bottom tissue culture dishes (MatTek) containing 2 mL of culture media, and their nuclei were stained with Hoechst 33342 (Invitrogen) according to manufacturer protocols ~10 min prior to imaging. D3 mouse embryonic stem cells (mESC) (#CRL-1934; ATTC, Manassas, VA, USA) were cultured in DMEM supplemented with 12.5% FBS, 0.1 mM Non-essential amino acids, 30 μg/mL Gentamicin (Gibco), 0.1 mM 2-mercaptoenthanol (Sigma-Aldridge, Oakville, ON, Canada) and 1000 U/ml leukemia inhibitory Factor (LIF) (EMD Millipore, Etobicoke, ON, Canada). Cells were passaged every 48 hours to prevent differentiation.

Isolation of 3T3 nuclei.

Cells at ~70% confluency were trypsinized, pelleted, and re-suspended in cold nuclei *ez* lysis buffer (Sigma-Aldridge, Oakville, ON, Canada). Samples were then spun down at 500 rpm for 5 min at 4°C, and re-suspended in ice cold *ez* storage buffer (Sigma-Aldridge, Oakville, ON, Canada), according to manufacturer's protocols. Isolated nuclei were stored at -80°C. Prior to experiments, nuclei were thawed and re-suspended in ice cold AFM buffer: protease inhibitor tablets (Roche), 1 mM EDTA (Fisher Scientific, Markham, ON, Canada) in 50 mL PBS (Fisher Scientific, Markham, ON, Canada). The isolated nuclei (in AFM buffer) were placed on Poly-L-Lysine (Sigma) coated petri dishes for 1 hour at 4°C, and Hoechst 33342 (Invitrogen, Burlington, ON, Canada) was added at a concentration of 1 μg/ml. This was followed by aspiration and fresh supply of AFM buffer (at room temperature). All experiments were performed immediately following.

Murine Models.

BALB/c mice, C57BL/10ScSnJ mice and Duchenne muscular dystrophic mouse model C57BL/10ScSn-Dmd^{mdx}/J were purchased from The Jackson Laboratory (Bar Harbor, ME,

USA). All animals were kept at constant room temperature (23°C) and humidity (78%) under a controlled light/dark cycle. Mice were fed a normal chow diet and were euthanized by CO_2 inhalation followed by cervical dislocation. All experimental procedures involving laboratory animals were approved by the Animal Care and Use Committee of the University of Ottawa.

Isolation of Myoblasts from Muscle Tissue.

The isolation of myoblast cells from the murine Duchenne muscular dystrophy model (*Dmd*^{mdx}) and its control murine model (C57BL/10) was based on the protocol of Li *et al.*, 2011 [1], and was performed as follows: Following euthanasia at 4 weeks of age, muscle tissues were resected from the lower mouse limbs in a sterile environment. The tissues were kept in PBS (Fisher Scientific, Markham, ON, Canada), minced into a coarse slurry using sterile scissors, and centrifuged for 5 min at 2500 rpm at 4°C. After a second wash/centrifugation, the tissue slurry was digested by a succession of protease treatment starting with collagenase (0.2%, 60 min at 37°C; Sigma-Aldridge, Oakville, ON, Canada), followed by dispase I (2.4 U/ml, 45 min at 37°C; Sigma-Aldridge, Oakville, ON, Canada) and ending with trypsin (0.25%, 20 min at 37°C; Wisent Bioproducts, St-Bruno, QC, Can). The cells were then re-suspended in DMEM (High glucose, HyClone, Logan, Utah, USA) containing 10% FBS (HyClone, Logan, Utah, USA), 10% horse serum (HyClone, Logan, Utah, USA), 1% newborn calf serum (HyClone, Logan, Utah, USA), 1% amphotericin B (Wisent Bioproducts, St-Bruno, QC, Canada), 1% P/S (Wisent Bioproducts, St-Bruno, QC, Canada) and 1% L-glutamine (Wisent Bioproducts, St-Bruno, QC, Canada). The cells were dissociated by passing the cell suspension 3 times through a 21G needle and filtered through a 70 µm cell strainer (Fisher Scientific, Markham, ON, Canada) to remove any larger debris. After a last centrifugation, the cells were plated in a collagen-coated T-25 flask for 2 hours at 37°C in a 5% CO_2 incubator. This first flask mainly contained fibroblasts and myofibroblasts. The non-adherent cells were then transferred into a second T-25 flask for 24 hours at 37°C in a 5% CO_2 incubator. This dish mainly contained myoblasts (confirmed by desmin staining, data not shown) and was used for experiments in the following days.

Isolation of Fibroblasts from Skin Tissue.

The isolation of skin fibroblasts from BALB/c mice was performed as follows: After euthanasia, the mouse dorsal skin was shaved and sterilized using 70% ethanol, and a 2 cm^2 dorsal skin section was resected. In a sterile environment, the skin was cut into small square sections (2

mm^2) and stored in PBS. These samples were then placed into Petri dishes, where physical contact between the skin samples and Petri dish surface were formed by positioning sterile glass coverslips over each sample. The samples were then immersed in DMEM (High glucose, HyClone, Logan, Utah, USA) containing 50% FBS (HyClone, Logan, Utah, USA), 1% amphotericin B (Wisent Bioproducts, St-Bruno, QC, Canada), 2% P/S and 1% L-glutamine (Wisent Bioproducts, St-Bruno, QC, Canada). When fibroblast cells migrated out of the skin (after 6-8 days), skin samples were then removed and the medium changed to normal growth media (DMEM: 10% FBS, 1% amphotericin B, 1% P/S and 1% L-glutamine).

Plasmids and Transfection.

D3 mESC and NIH 3T3s were transfected with lamin A-GFP plasmids (a kind gift of Tom Rapoport, Harvard University) using Lipofectamine 2000 (Invitrogen, Burlington, ON, Canada) according to manufacturer protocols. Transfections were done 24 hours after cell passage, 24 hours prior to experiments.

Immunofluorescence.

Staining for actin, microtubules (MTs) and DAPI was achieved following a previously reported protocol [2]. In brief, cells were first rinsed with warm PBS (Fisher Scientific, Markham, ON, Canada) and fixed with a solution of 2% sucrose and 3.5% paraformaldehyde (Fisher Scientific, Markham, ON, Canada). They were permeabilized with warm 0.5% Triton X-100 (Fischer). Samples were incubated with Phalloidin Alexa Fluor 546 (Invitrogen, Burlington, ON, Canada) to stain for actin. MTs were stained on ice by first incubating with monoclonal (mouse) anti-α-tubulin (Sigma-Aldridge, Oakville, ON, Canada), then with Alexa Fluor 488 conjugated rabbit anti-mouse immunoglobins (Invitrogen, Burlington, ON, Canada) for 15 min with a 15 min wash following each incubation. Lastly, nuclei were labelled by incubating with DAPI (Invitrogen, Burlington, ON, Canada). Staining for lamin-A in mESC was achieved using a mouse anti-lamin-A (Abcam, Toronto, ON, Canada) primary antibody.

Drug Treatments.

In order to study the influence of the cytoskeleton on nuclear deformation, cells were treated with either Cytochalasin-D (CytD) (10 µM in DMSO, Sigma-Aldridge, Oakville, ON, Canada) or nocodazole (Noco) (10 µM in DMSO, Sigma-Aldridge, Oakville, ON, Canada) to specifically depolymerize actin or tubulin, respectively. Double-drug experiments, in which the cells were

treated with both of these drugs (NocoCytD) simultaneously, were also performed. Appropriate amounts of each drug were added to the culture media 15 min prior to the experiment or, in the case of staining, 15 min prior to fixing. In order to examine the role of chromatin organization, cells were treated with trichostatin A (TSA, Sigma-Aldridge, Oakville, ON, Canada), a known histone deacetylase inhibitor [3,4]. Cells were treated with 150 ng/mL TSA solubilized in DMSO for 24 hours prior to experimentation/imaging.

Simultaneous Optical and Atomic Force Microscopy.

Atomic Force Microscopy (AFM) and high-speed laser scanning confocal microscopy (LSCM) were performed simultaneously by mounting the AFM (NanoWizard II, JPK Instruments AG, Berlin, Germany) onto a Nikon TiE inverted microscope (Nikon, Melville, NY, USA) with a resonant scanner A1-R confocal. A 60X, NA 1.2 water immersion objective was used for all experiments, and the culture dish was kept at 37°C with a temperature-controlled stage. All pyramidal tipped AFM cantilevers had an experimentally determined stiffness, $k = 0.06 \pm 0.01$ N/m (MSCT-AUHW; Veeco, Plainview, NY, USA). For large-contact forces, spherical polystyrene beads (10 µm, Fluka Sigma-Aldridge, Oakville, ON, Canada) were glued to an AFM cantilever (PNP-TR-50, Nanoworld, Neuchâtel, Switzerland) using a UV-curing optical adhesive (Norland Products, Cranbury, NJ, USA) as in the JPK technical notes (found at http://www.jpk.com/afm.230.en.html).

Tracking nuclear deformation entailed capturing images in a single plane (x-y) set in the middle of the nucleus at a rate of 4 frames/s. Images were recorded through 20 s of constant force application (contact mode), as well as 2.5 s before extension of the AFM tip as control. During imaging, the AFM cantilever was brought into contact with the cell surface, above the centre of the nucleus, and a constant force was applied. Only the first 5 s of data was used for analysis to prevent focal drift. Long-term nuclear deformation (15 min) was recorded by performing simultaneous AFM and 4D LSCM. Image volumes of the nucleus were recorded 1 min prior to force application and once every min for 15 min thereafter. As before, the AFM cantilever was aligned over the centre of the cell nucleus and used to apply a constant force of 10 nN or 20 nN for 15 min while the structural deformation of the nucleus was recorded. Only single cells were analyzed, except in the case of some primary cells and mESCs where islands of cells were unavoidable. In these cases, only cells at the periphery of islands were used for deformation

experiments. We have previously shown that cells respond to highly localized deformations in a similar manner, whether grown as single cells or within a monolayer[5].

Image Analysis.

Following AFM/LSCM experiments, raw image data was analysed using ImageJ [6] in order to quantify the deformation of the nucleus. A small macro was generated in order to automate the analysis. For long-term (15 min) deformation analysis, maximum intensity projections of image volumes of nuclei were acquired at each time point. Each resulting image was converted into a corresponding binary mask, which allowed the border of the nucleus to be selected and the area automatically determined at each time point. Simultaneously, each selection was fitted to an ellipse, from which we obtained the lengths of the major and minor axes. The method for processing short-term (5 s) deformation was very similar but did not require a maximum intensity projection at each time point. Typically, mESC transfected with lamin-A EGFP appeared as clusters of embryoid bodies, and so the green fluorescent channel was used to fit the nuclear ellipse instead of the blue fluorescent channel (Hoechst, DNA).

Statistical Tests and Fitting Data.

All statistical analyses were performed using Origin 8.5. For all experiments, the response of cells was averaged for each condition (untreated, CytD, Noco and NocoCytD) and for each force (0 nN, 10 nN and 20 nN). In total ~75% of cells displayed behaviour consistent with the data presented. In a minority of cases nuclei did not deform or move during the measurement and were excluded from the analysis. All data presented are mean ± s.e.m. Statistical significance is considered for P-values where $P < 0.05$, as determined by two-tailed student's t-tests, unless noted otherwise.

Plateau strains in both the major and minor nuclear axes were obtained by fitting the time-dependent strain data (see Fig. 1, in main text) to a viscoelastic Kelvin-Voigt model:

$$\varepsilon(t) = \varepsilon_p\left(1 - e^{-t/\tau}\right) \quad (1)$$

where $\varepsilon(t)$ is the time-dependent strain, ε_p represents the plateau strain observed in the long duration elastic regime, and τ is the characteristic deformation time constant which is inversely

proportional to the deformation rate ($\kappa = \tau^{-1}$). From the fits, we extracted $\varepsilon_{p\ major}$ and $\varepsilon_{p\ minor}$ for the major and minor axis, respectively. Average R^2 values were > 0.90 on average.

Characterization of Nuclear Shape Change.

Change in nuclear shape was examined by measuring circularity (Circ) using a built-in shape descriptor in ImageJ, measured as:

$$Circ = (4\pi \times Area)/Perimeter^2 \quad (2)$$

A value of 1.0 represents a perfect circle, whereas values approaching zero signify an elliptical shape.

Thymidine Block for Examination of Cell Cycle.

To investigate whether nuclear anisotropy would still be observed at different phases during the cell cycle, we employed a double thymidine block, as in [7], in order to arrest 3t3s in the G1/S, and G2/M phases. Cells were treated with 2 mM Thymidine (Calbiochem, Etobicoke, ON, Canada) for 18 hours, followed by a release for 9 hours by replacing with complete media. Cells were then blocked again, with 2 mM Thymidine for another 17 hours, prior to performing experiments on cells (in the G1/S transition phase). For cells in the G2/M transition phase, the block was released for another 10 hours, prior to commencement of the second experiment. Three separate experiments were performed, with experiments performed on cells in the G1/S and G2/M transition phases on the same day, from cells of the same population, and in one case also with untreated cells.

References

1. Li, Y., Pan, H. & Huard, J. Isolating stem cells from soft musculoskeletal tissues. *Journal of visualized experiments: JoVE*, (2010).

2. Pelling, A. E., Veraitch, F. S., Chu, C. P. K., Mason, C. & Horton, M. A. Mechanical dynamics of single cells during early apoptosis. *Cell Motil. Cytoskeleton* 66, 409-422, (2009).

3. Furumai, R. *et al.* Potent histone deacetylase inhibitors built from trichostatin a and cyclic tetrapeptide antibiotics including trapoxin. *Proc. Natl. Acad. Sci. USA* 98, 87-92, (2001).

4. Yoshida, M., Kijima, M., Akita, M. & Beppu, T. Potent and specific-inhibition of mammalian histone deacetylase both invivo and invitro by trichostatin-a. *J. Biol. Chem.* 265, 17174-17179, (1990).

5. Haase, K. & Pelling, A. E. Resiliency of the plasma membrane and actin cortex to large-scale deformation. *Cytoskeleton* 70, 494-514, (2013).

6. Abramoff, M. D., Magelhaes, P. J. & Ram, S. J. Image processing with imagej. *Biophotonics international* 11, 36-42, (2004).

7. Mori, S. *et al.* Myb-binding protein 1a (mybbp1a) is essential for early embryonic development, controls cell cycle and mitosis, and acts as a tumor suppressor. *Plos One* 7, (2012).

Supplementary Figures

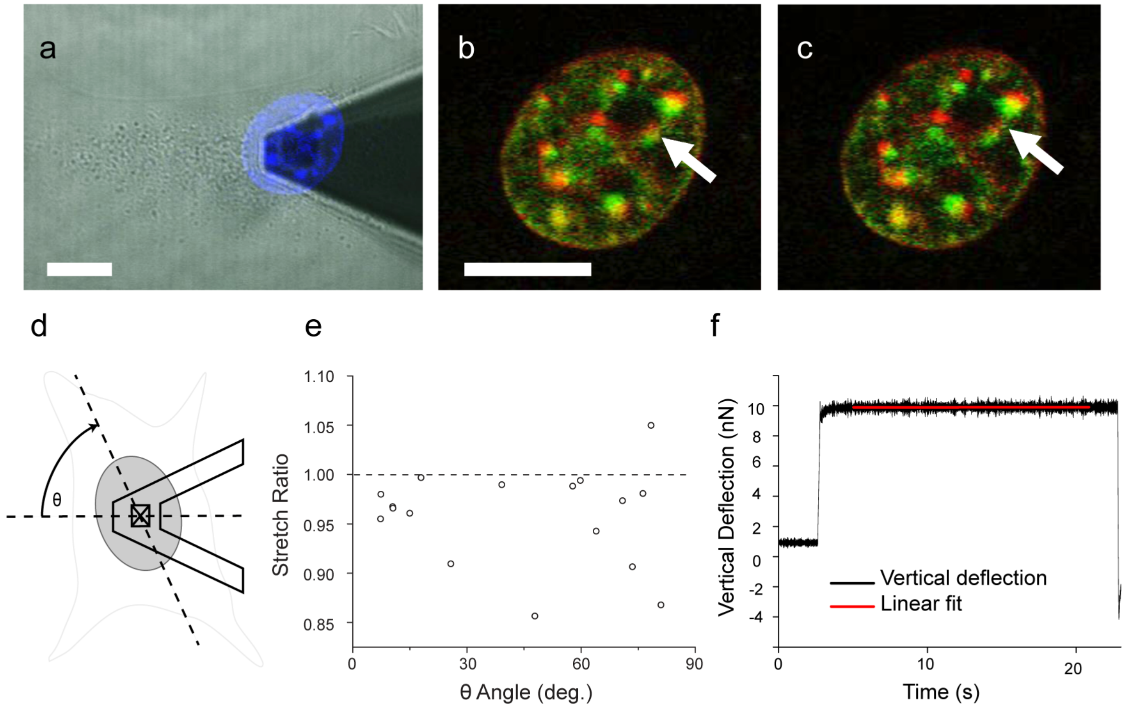

Figure S1. Nuclear expansion is evident and not affected by AFM tip orientation.

(a) Transmitted light image of 3T3 nuclei during a 10 nN load, applied by AFM. Blue = DNA. The nucleus does not move out of the imaging plane due to mechanical loading, see Vid. S1. and image overlays of 0 nN (red) and 10 nN (green) following (b) 0.25 s, and (c) 5 s of force. Movement of small sub-nuclear components occurs following nuclear expansion; however large nucleoli (arrows) remain stationary during the applied stress, indicating no substantial out-of-plane motion. Scale bar is 10 μm. (d) Schematic showing the AFM tip as pyramidal with a square base in relation to the position of the nucleus. (e) To ensure that the orientation of the tip with respect to nuclear orientation had no influence on the observed stretch ratio, we calculated the angle the long nuclear axis formed with the long axis of the AFM cantilever (n = 16 cells). Nuclei were always randomly oriented between 0-90 degrees in all experiments, and no effort was made to choose cells oriented in a particular fashion. The orientation of the nucleus had no clear influence on the observed anisotropy as revealed in the plot of SR versus orientation angle. (f) Cantilever deflection sampled at 1kHz during an application of 10nN to intact 3T3 cells. The applied force is very stable over time, only a very small standard deviation of 0.18nN was observed in the linear region (red).

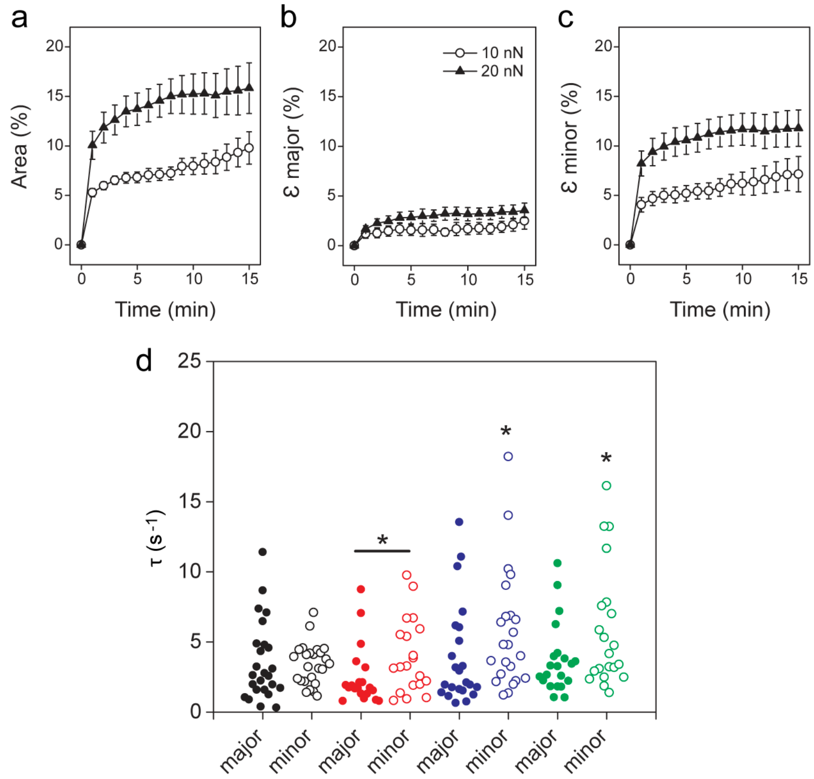

Figure S2. Nuclear anisotropy is observed over long-term deformations and is influenced by actin.

(a) Projected nuclear area following 15 min of 10 nN (white circle) and 20 nN loads (black triangle). Nuclear expansion is clearly dependent on force magnitude (*P* < 0.001). Projected area increased by 7.2 ± 1.6% and 14.1 ± 2.1% following 15 min of a 10 nN (n = 12) and 20 nN (n = 11) constant force, respectively. Plots of force-dependent strain: along the (b) major axis and (c) minor nuclear axis as a function of time. Cells experienced highly anisotropic nuclear strains of ε_{major} = 1.6 ± 0.7% versus ε_{minor} = 5.6 ± 1.5% when exposed to 10 nN, and larger strains of ε_{major} = 2.6 ± 1.1% versus ε_{minor} = 11.3 ± 2.0% when exposed to 20 nN. Change in area and strain are dependent on the magnitude of the force applied, however, anisotropy is not. Error bars are s.e.m. (d) Actin influences the characteristic deformation time constant. Distribution of deformation time constants ($\tau = s^{-1}$) for untreated (black, n=25), and treated cells: Noco (red, n=20), CytD (blue, n=23) and NocoCytD (green, n=21), as determined by fits of the strain data to equation (1). Characteristic relaxation time constants (untreated: τ_{major} = 3.41 ± 0.29 s^{-1}, and τ_{minor} = 10.78 ± 7.24 s^{-1}) display a significant amount of variance, and so there is no dependence between major and minor axes, except for the case of Noco treated cells (P < 0.05). However, time constants for

cells treated with CytD (5.73 ± 0.88 s^{-1}) and NocoCytD (5.87 ± 0.94 s^{-1}) along the minor axis, were significantly ($P < 0.02$) increased in comparison to untreated 3t3s. Loss of an intact actin network clearly resulted in a faster deformation along the minor nuclear axis.

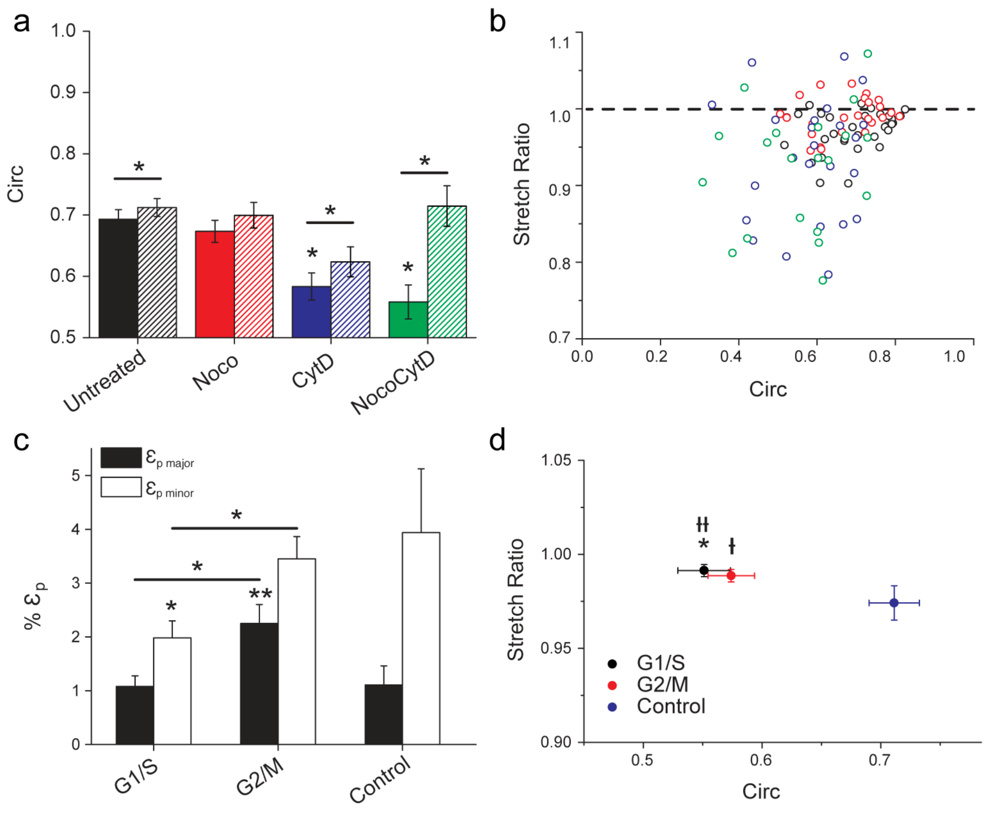

Figure S3. Cell shape is not correlated with anisotropy, but is affected by cell cycle.

(a) Change in circularity is demonstrated in the absence of loading (solid bars) and following a 10 nN load (hatched bars). Treatment with CytD (blue) and NocoCytD (green) resulted in significantly increased change in circularity following loading (0 nN), in comparison to untreated 3T3s (black), and Noco (red), as measured by [2]. (*) Indicates significance ($P < 0.05$) with two sample t-test, while (* with bar) indicates significance ($P < 0.05$) with paired t-test between measures of circularity for 0 nN and 10 nN. (b) Plot of stretch ratio and circularity for untreated, and treated 3t3s. There is no correlation between initial cell shape (0 nN) and the magnitude of observed anisotropy. (c) Mean plateau strains (ε_p) are shown for cells in the G1/S (n=30) and G2/M (n=36) transition phases, in comparison to non-blocked cells from the same population (n=15). Nuclear strain is significantly greater in both axes, in response to force, in G2/M in comparison with those cells blocked in the G1/S phase. Significance (without bars) is shown

with respect to untreated 3T3s. (d) Mean anisotropic stretch ratio and circularity is shown for untreated and cell-cycle blocked 3T3s. Anisotropy was reduced for cells blocked in the G1-S phase. Cell shape was also affected, resulting in less circular nuclei. (* $P < 0.05$, with t-test for SR, and † $P < 0.001$, †† $P < 0.0001$, with t-test for circularity). Shown is mean ± s.e.m

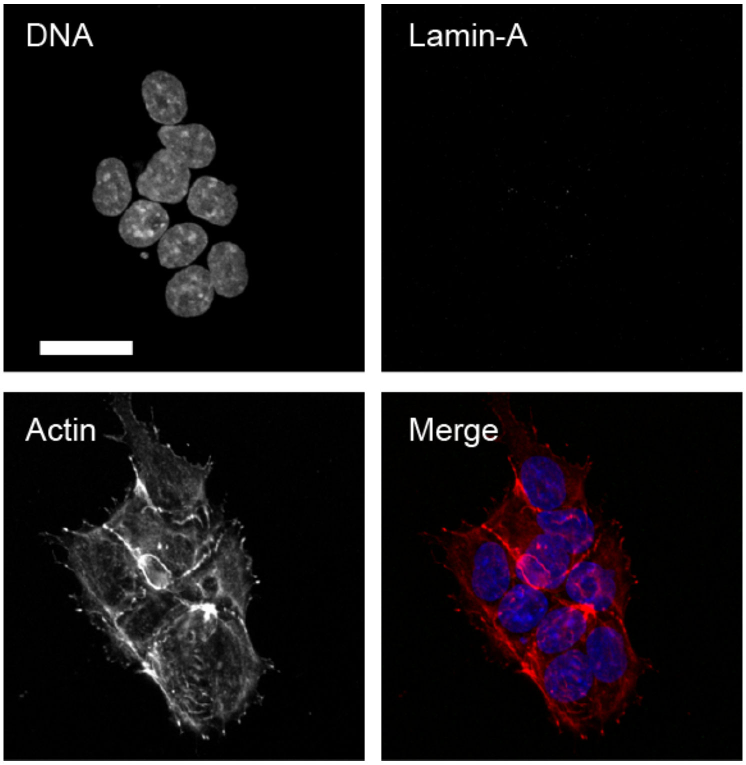

Figure S4. **Immunofluorescent images of D3 mESC demonstrate their lack of lamin-A.** D3 stem cells were stained for DNA (blue), lamin-A (green), and actin (red). Only a very faint fluorescent signal is observed for lamin-A in mESC. Scale bar is 25 μm.

Supplementary Tables

Table S1. The cytoskeleton affects nuclear strain and anisotropy

NIH 3T3	N	$\varepsilon_{p\ major}$ (%)	$\varepsilon_{p\ minor}$ (%)	SR($\lambda_{major}/\lambda_{minor}$)	Circ	Area (μm^2)
Isolated	23	2.8 ± 0.9	8.0 ± 1.4*	0.95 ± 0.01	0.53 ± 0.02*	102.8 ± 3.9
Untreated	32	1.7 ± 0.3	4.7 ± 0.5	0.97 ± 0.00	0.69 ± 0.02	140.6 ± 5.3
Noco	24	3.6 ± 0.5*	4.6 ± 0.5	0.99 ± 0.00*	0.67 ± 0.02	130.9 ± 7.2
Cytd	24	7.7 ± 1.5*	15.7 ± 1.7*	0.93 ± 0.02*	0.58 ± 0.02*	134.7 ± 4.1
NocoCytd	21	7.7 ± 1.1*	19.4 ± 2.9*	0.91 ± 0.02*	0.56 ± 0.03*	142.2 ± 5.7
Bead	26	3.9 ± 0.5	8.4 ± 0.8	0.96 ± 0.01	0.51 ± 0.03*	-

Using equation (1), we determined the plateau strains (ε_p) for the major and minor nuclear axes. In all cases, except for Noco-treated cells, $\varepsilon_{p\ minor}$ was significantly ($P < 0.05$, paired t-test) greater than $\varepsilon_{p\ major}$. The mean stretch ratio (SR) was < 1 in all cases, indicative of anisotropic nuclear expansion. All values listed are for nuclei within intact cells, except for "Isolated". "Bead" refers to the same experiment performed on untreated 3t3s with a spherical AFM tip, instead of a pyramidal one. The difference in circularity between the experiment using the bead and pyramidal tip is most likely due to normal variance across samples (see Fig. S3B) (* indicates $P < 0.05$ significance with t-test). See also Fig. 3 in main text. Values shown are mean ± s.e.m.

Table S2. Nuclear strain is anisotropic in established and primary cells

	N	$\varepsilon_{p\ major}$ (%)	$\varepsilon_{p\ minor}$ (%)	SR ($\lambda_{major}/\lambda_{minor}$)	Circ (0nN)
NIH 3T3	32	1.7 ± 0.3	4.7 ± 0.5	0.97 ± 0.00	0.69 ± 0.02
HFF	32	2.1 ± 0.4	3.9 ± 0.6	0.98 ± 0.00	0.52 ± 0.03
CHO	17	2.9 ± 0.4	4.7 ± 0.6	0.98 ± 0.00	0.63 ± 0.02
MDCK	30	1.8 ± 0.2	4.3 ± 0.4	0.98 ± 0.00	0.55 ± 0.02
HeLa	26	2.0 ± 0.3	5.1 ± 0.7	0.97 ± 0.01	0.48 ± 0.02
C2C12	20	1.6 ± 0.3	4.2 ± 0.5	0.98 ± 0.00	0.56 ± 0.03
BALB/c fibro	15	3.0 ± 0.7	9.6 ± 1.3	0.94 ± 0.01	0.44 ± 0.03
D3 mESC	10	5.6 ± 0.8	8.0 ± 1.7	0.98 ± 0.01	0.52 ± 0.05
C57BL/10 myob	16	1.4 ± 0.5	5.5 ± 0.7	0.96 ± 0.01	0.63 ± 0.03
Dmd^{mdx} myob	9	4.9 ± 1.1	5.5 ± 1.0	0.99 ± 0.01	0.50 ± 0.03
C57BL/10 fibro	30	1.8 ± 0.3	3.9 ± 0.6	0.98 ± 0.01	0.66 ± 0.03
Dmd^{mdx} fibro	22	2.5 ± 0.6	4.0 ± 0.9	0.99 ± 0.01	0.62 ± 0.02

Mean plateau strains (ε_p) in the minor axis were significantly greater than the major axis for all cell types ($P < 0.05$, paired t-test), except for mESC ($P = 0.11$) and Dmd myoblasts ($P = 0.42$). Population means of SR are significantly different, but variances are not (using one-way ANOVA with Tukey and Levene's test with $P < 0.05$). Values shown are mean ± s.e.m.

Table S3. Cell cycle affects nuclear area

Expt	N	Area (μm^2) 0nN	Area (μm^2) 10nN	% Area change
G1/**S**	16	229.6 ± 13.8	234.0 ± 13.7	2.1 ± 0.5*
M/**G2**	18	180.0 ± 11.5	177.1 ± 14.6	0.8 ± 5.7
G1/**S**	15	284.4 ± 21.6	294.7 ± 20.4	4.4 ± 1.0*
M/G2	17	297.9 ± 28.9	309.7 ± 28.3	4.8 ± 1.2*
G1/**S**	16	213.8 ± 10.3	219.6 ± 9.5	3.1 ± 0.7*
M/**G2**	16	212.5 ± 15.5	224.3 ± 15.5	6.2 ± 1.2*

Cells blocked by thymidine in the G1/S and M/G2 phases resulted in decreased circularity (Fig. S3 d), but increased area on average (* indicates $P < 0.05$ significance with paired t-test). Values shown are mean ± s.e.m for 3 separate experiments.
